# Alzheimer disease-associated tau post-translational modification mimics impact tau propagation and uptake

**DOI:** 10.1093/jnen/nlaf007

**Published:** 2025-02-21

**Authors:** John R Dickson, Robert G R Sobolewski, Analiese R Fernandes, Joanna M Cooper, Zhanyun Fan, Mirra Chung, Cameron Donahue, Derek H Oakley, Dudley K Strickland, Bradley T Hyman

**Affiliations:** Alzheimer Research Unit, Department of Neurology, Massachusetts General Hospital, Charlestown, MA, United States; Faculty of Medicine, Harvard Medical School, Boston, MA, United States; Alzheimer Research Unit, Department of Neurology, Massachusetts General Hospital, Charlestown, MA, United States; Alzheimer Research Unit, Department of Neurology, Massachusetts General Hospital, Charlestown, MA, United States; The Center for Vascular and Inflammatory Diseases, University of Maryland School of Medicine, Baltimore, MD, United States; Department of Physiology, University of Maryland School of Medicine, Baltimore, MD, United States; Alzheimer Research Unit, Department of Neurology, Massachusetts General Hospital, Charlestown, MA, United States; Alzheimer Research Unit, Department of Neurology, Massachusetts General Hospital, Charlestown, MA, United States; Alzheimer Research Unit, Department of Neurology, Massachusetts General Hospital, Charlestown, MA, United States; Faculty of Medicine, Harvard Medical School, Boston, MA, United States; Department of Pathology, C.S. Kubik Laboratory for Neuropathology, Massachusetts General Hospital, Boston, MA, United States; The Center for Vascular and Inflammatory Diseases, University of Maryland School of Medicine, Baltimore, MD, United States; Department of Physiology, University of Maryland School of Medicine, Baltimore, MD, United States; Department of Surgery, University of Maryland School of Medicine, Baltimore, MD, United States; Alzheimer Research Unit, Department of Neurology, Massachusetts General Hospital, Charlestown, MA, United States; Faculty of Medicine, Harvard Medical School, Boston, MA, United States

**Keywords:** acetylation, Alzheimer disease, low-density lipoprotein receptor-related protein 1, phosphorylation, post-translational modification, propagation, tau

## Abstract

As Alzheimer disease (AD) progresses, pathological tau spreads by cell-to-cell propagation of tau. This study aims to elucidate the impact of AD-associated post-translational modifications of tau-on-tau propagation. Tau propagation reporter constructs distinguishing donor cells from recipient cells were developed, and additional constructs were made with tau residues mutated from serine or threonine to aspartate to mimic the negative charge of a phosphorylation and/or from lysine to glutamine to mimic the charge-neutralizing effect of acetylation. Flow cytometry was used to quantify donor and recipient cells. This revealed that the mutations generally tended to reduce tau propagation compared to wildtype tau. Recombinant tau containing either wildtype or posttranslational modification mimicking mutations were used to treat Chinese hamster ovary cells or human induced pluripotent stem cell-derived neurons to quantify tau uptake, revealing that the mutations generally resulted in reduced uptake compared to wildtype tau. Surface plasmon resonance revealed that the mutations had a reduced affinity for lipoprotein receptor-related protein 1 (LRP1), a tau uptake receptor, compared to wildtype tau. Overall, these results suggest that AD-associated posttranslational modification mimicking mutations reduce the cell-to-cell propagation of tau by reducing tau uptake by recipient cells, which may be in part due to reduced binding affinity to LRP1.

## INTRODUCTION

Alzheimer disease (AD) is a neurodegenerative disorder characterized clinically by progressive cognitive decline,^1^ and neuropathologically by progressive accumulation of amyloid-β and tau aggregates.^2^ Tau aggregates in the form of neurofibrillary tangles display a stereotyped progressive accumulation in specific neuroanatomical regions as defined by the Braak stages.^3–5^ The cellular mechanism underlying this phenomenon is thought to be the cell-to-cell propagation of pathological tau across neural circuits, followed by templated misfolding to amplify tau in the recipient cell.^6^ The propagation of tau occurs when a donor cell releases tau, and this tau is subsequently taken up by a recipient cell.^7^ In cell culture models, the release of tau can occur generally into the culture medium,^7^ or in the case of neurons, it can occur through synaptic interactions.^8^ In vivo, the trans-synaptic propagation of tau across neuroanatomically connected neuronal circuits has also been observed.^9^ Proposed mechanisms of release from donor cells include secretion through the plasma membrane, extracellular vesicles, and exocytosis.^6^ For tau uptake by recipient cells via endocytosis, low density lipoprotein receptor-related protein 1 (LRP1) is the canonical tau uptake receptor.^10^^,^^11^ A structurally similar protein, sortilin-related receptor 1, has also been identified as a tau uptake receptor.^12^ A recent study has also suggested an endocytosis-independent mechanism of tau entry into recipient cells, possibly resulting from direct translocation across the cell membrane.^13^ Given its link to the spread of tau pathology in the brains of patients with AD, tau propagation is of interest for both understanding AD pathophysiology as well as considering therapeutic approaches to slow or stop the progression of disease.^6^

Not surprisingly, the properties of tau can influence its propagation from cell to cell. For example, posttranslational modifications of tau can affect aspects of tau propagation. In a study of tau propagation following intracerebral injection of AD-derived tau in the hippocampus of hTau mice, in vitro dephosphorylation of AD-derived tau led to significantly reduced tau propagation compared to phosphorylated tau.^14^ Although those experiments were performed in the hTau mouse model, which expresses human tau,^15^ other experiments have demonstrated tau propagation on a tau null background, suggesting that propagating tau does not require endogenous tau for templated seeding in order to propagate.^16^ For the tau-LRP1 interaction, lysine residues are critical for promoting this interaction, and chemically capping tau’s lysine residues prevents LRP1-mediated tau uptake.^10^ A biological modification of lysine residues can occur as a posttranslational modification by acetylation, and the charge neutralization by lysine acetylation can be mimicked with a lysine to glutamine mutation (pseudoacetylation). Making these pseudoacetylation mutations at certain residues in tau reduces LRP1 binding and tau uptake.^11^ Another property of tau that is related to propagation is the oligomerization status of tau. Through size exclusion chromatography, AD-derived tau can be separated into low molecular weight (LMW) tau monomers or high molecular weight (HMW) tau oligomers. The HMW tau oligomers are readily taken up into neurons, whereas the LMW tau monomers are not.^8^ However, it is not clear whether this phenomenon is caused by the size of tau or an associated feature, such as posttranslational modifications (PTMs). Through extensive mass spectrometric analysis, the posttranslational modifications of LMW and HMW tau species have been comprehensively identified; this analysis demonstrated that tau in HMW oligomers is more extensively post-translationally modified compared to LMW tau species.^17^ Since PTMs can alter a protein’s function, we wondered if AD-associated tau PTMs could impact the cell-to-cell propagation of tau.

This study aims to understand the influence of mutations mimicking AD-associated tau PTMs found in LMW and HMW tau oligomers on tau propagation. Our approach is to use cell-based and biochemical assays to study the effect of defined mutations mimicking PTMs of tau-on-tau propagation, release, uptake, and interaction with LRP1. Our overall hypothesis is that some AD-associated tau PTM mimics will alter tau propagation through altered tau release, uptake, or both.

## METHODS

### Ethics approval

Ethics approval for fibroblast donation was provided by the Mass General Brigham Institutional Review Board (1999P003693).

### Plasmid cloning

The majority of the mammalian expression plasmids based on pcDNA3.1 containing the WT or multiple mutant tau sequences were custom prepared by Biomatik (Kitchener, ON, Canada). Single point mutations were prepared by using the WT tau propagation construct with an amino-terminal V5 tag as a basis. Custom DNA fragments containing the desired single mutations were prepared by Twist Bioscience (South San Francisco, CA). For mutations at amino acid positions lower than 300, the fragments were cloned into the base vector between *pmL1* and *BstEII* restriction sites. For mutations at amino acid positions at 300 or higher, the fragments were cloned into the base vector between *BstEII* and *EcoRV* restriction sites. A similar approach was taken for cloning the tau mutant constructs that prevent phosphorylation and acetylation (8A2R and 20A3R), with custom DNA fragments containing the desired mutations obtained from Twist Biosciences. The 20D3Q tau propagation construct that mimics the phosphorylations and acetylations found on HMW tau was used as the basis for cloning, and the fragments were cloned into the base vector between *EcoRV* and *BsiW1* restrictions sites. In all cases, cloning was performed using the NEBuilder HiFi DNA Assembly Cloning Kit (New England Biolabs, Ipswich, MA).

For bacterial expression plasmids without a HiBiT-tag, a pre-existing pET28 vector was used as the base vector. The tau propagation constructs as described above were used to derive the desired tau sequences by restriction digest at *Nde1* and *Xho1* restriction sites for the WT sequence and *BsiWI* and *Nhe1* restriction sites for the 8D2Q and 20D3Q sequences. The insert fragments were cloned into the base vector at the same restriction sites. For bacterial expression plasmids with a HiBiT-tag, the HiBiT-tag was inserted using the previously described untagged bacterial expression plasmids as base vectors. A fragment containing the desired tag sequence as a custom order from Twist Biosciences was obtained and cloned into the base vectors between *BgLII* and *BsiW1* restriction sites.

The sequence of all plasmids was confirmed by rapid plasmid sequencing at the Massachusetts General Hospital DNA Core.

### Chinese hamster ovary (cell culture and transfection)

Chinese hamster ovary (CHO) cells were cultured in DMEM/F-12 with GlutaMAX supplement (ThermoFisher Scientific, Waltham, MA) supplemented with 10% fetal bovine serum (ThermoFisher Scientific) and 1% Penicillin-Streptomycin (ThermoFisher Scientific). The cells were incubated at 37 °C with 5% CO_2_. The CHO cells were transfected with Lipofectamine 2000 (ThermoFisher Scientific). Media exchange was performed 4 hours after transfection.

### Induced pluripotent stem cell culture

Induced pluripotent stem cells (iPSCs) stably expressing a doxycycline-driven human neurogenin 2 (hNGN2) expression cassette^18^ were obtained from the Massachusetts Alzheimer’s Disease Research Center Neuropathology Core. The iPSCs were cultured in mTeSR Plus media (StemCell Technologies, Vancouver, Canada) on Matrigel-coated dishes (Corning Inc., Corning, NY).

### Neuronal differentiation

Neuronal differentiation of iPSCs was performed by doxycycline driving expression of hNGN2. On day in vitro (DIV)-1, the iPSCs were dissociated with Accutase (Corning, Inc.) and plated on Matrigel-coated plates in mTeSR Plus media plus 1 µM thiazovivin (Millipore Sigma, Burlington, MA). On DIV 0, the media was exchanged to DMEM/F12 media containing Glutamax-I (ThermoFisher Scientific), non-essential amino acids (ThermoFisher Scientific), N-2 supplement (ThermoFisher Scientific), 10 ng/mL brain-derived neurotrophic factor (BDNF) (R&D Systems, Minneapolis, MN), 10 ng/mL NT-3 supplement (ThermoFisher), and 2 µg/mL doxycycline (Millipore Sigma). On DIV 2, the media was exchanged with DMEM/F12 media containing Glutamax-I and supplemented with B-27 supplement (ThermoFisher Scientific), 10 ng/mL BDNF, 10 ng/mL NT-3 supplement, and 2 µg/mL doxycycline. On DIV 4, the cells were passaged onto poly-D-lysine plates (Corning Inc.) coated with Matrigel. On DIV 5, the media was changed to Neuro-basal media (ThermoFisher Scientific) supplemented with B-27 supplement, 10 ng/mL BDNF, 10 ng/mL NT-3 supplement, 10 ng/mL glial cell line-derived neurotrophic factor (GDNF) (R&D Systems), 2 µg/mL doxycycline, 2% horse serum (ThermoFisher Scientific), 5 µg/mL puromycin (ThermoFisher Scientific). Starting on DIV 7, 50% of the media was exchanged every 2 days until DIV 14.

### Antibodies

The antibodies in the Table 1 were stored according to the manufacturer’s instructions. Dilutions used in experiments are included in the Table 1.

**Table 1.** Antibody list.

Antibody	Host	Application	Dilution	Manufacturer	Catalog #
V5 epitope tag	Chicken	ICC	1:1000	abcam	ab9113
V5-AF488	Rabbit	Flow cytometry	1:275	Biotechne	FAB8926G
HT7	Mouse	ICC	1:1000	ThermoFisher	MN1000
HT7-AF488	Mouse	Flow cytometry	1:275	ThermoFisher	53-5916-42
HiBiT	Mouse	ICC	1:1000	Promega	N7200
α-Tubulin	Rabbit	ICC	1:1000	abcam	ab52866
MAP2	Rabbit	ICC	1:1000	abcam	ab32454
Anti-Chicken IgY (H + L), AF488	Goat	ICC	1:1000	ThermoFisher	A-11039
Anti-Mouse (H + L), AF488	Goat	ICC	1:1000	ThermoFisher	A28175
Anti-Rabbit (H + L), AF647	Goat	ICC	1:1000	ThermoFisher	A-21244
Tau	Rabbit	Western blot	1:5000	DAKO	A0024
His epitope tag	Mouse	Western blot	1:1000	ThermoFisher	PA1-983B
IRDye 680RD anti-Mouse IgG	Donkey	Western blot	1:5000	LICORbio	926-68072
IRDye 800CW anti-Rabbit IgG	Donkey	Western blot	1:5000	LICORbio	926-32213

### Immunocytochemistry

Cells were washed twice with phosphate-buffered saline (PBS) (ThermoFisher Scientific) and fixed in 4% paraformaldehyde (ThermoFisher Scientific) in PBS for 15 minutes. The cells were then washed with PBS and permeabilized with 0.2% triton X-100 (Millipore Sigma) in PBS for 15 minutes followed by a PBS wash. Blocking was performed with 10% normal goat serum in 0.02% triton X-100 in PBS for 30 minutes at room temperature. The incubation with primary antibodies diluted in 5% normal goat serum in 0.02% triton X-100 in PBS was performed at 4 °C overnight. The cells were washed 3 times with PBS. The incubation with secondary antibodies diluted in 5% normal goat serum in 0.02% triton X-100 in PBS was performed at room temperature for 1 hour. Nuclei were counterstained with 4’,6-diamino-2-phenylindole (DAPI) by coverslipping with Fluoromount-G Mounting Medium with DAPI (ThermoFisher Scientific).

### Microscopy

Confocal microscopy was performed using a FLUOVIEW FV3000 Confocal Laser Scanning Microscope (Olympus, Tokyo, Japan). Images were acquired using a 40× air objective.

### Flow cytometry

In preparation for flow cytometry, cells in a 96-well plate were treated with trypsin for 5 minutes at 37 °C and suspended in cell culture media with pipetting up and down to produce a single cell suspension. The cells were transferred to a round-bottom 96-well plate for subsequent centrifugation steps. The cells were prepared for intracellular antibody staining with the eBioscience Intracellular Fixation & Permeabilization Buffer Set (ThermoFisher Scientific). The antibodies used for intracellular staining were the V5 Epitope Tag Alexa Fluor 488-conjugated Antibody (R&D Systems) or Tau Monoclonal Antibody (HT7), Alexa Fluor 488 (ThermoFisher Scientific). Flow cytometry was performed with a MACSQuant VYB flow cytometer (Miltenyi Biotec, Bergisch Gladbach, Germany).

### Enzyme-linked immunosorbent assay

The media from transfected cells was removed for analysis. The cells were washed with PBS, and then the cells were lysed with M-PER Mammalian Protein Extraction Reagent (ThermoFisher Scientific) containing 1X Halt Protease and Phosphatase Inhibitor Cocktail (ThermoFisher Scientific). Prior to the enzyme-linked immunosorbent assay (ELISA), the media was diluted 1:10 in PBS containing 1X Halt Protease and Phosphatase Inhibitor Cocktail, and the cell lysate was diluted 1:100 in PBS containing 1X Halt Protease and Phosphatase Inhibitor Cocktail. Tau quantification was performed with the R-PLEX Human Tau (total) Assay (Meso Scale Discovery Diagnostics, Rockville, MD).

### Protein expression and purification

Transformation of BL21 Competent *E. coli* cells (ThermoFisher) with bacterial expression plasmids was performed with heat shock. Transformed cells were plated on a Luria broth-agar plate containing 50 µg/mL kanamycin as the selection antibiotic and incubated overnight at 37 °C. A 3 mL starter culture of Luria broth containing kanamycin was inoculated with a single colony and incubated overnight at 37 °C. A 1 L culture of Luria broth containing kanamycin was inoculated with 1 mL of the starter culture and incubated at 37 °C with monitoring of the optical density at 600 nm (OD600) until reaching 0.6. Expression was induced with isopropyl β-D-1-thiogalactopyranoside (IPTG) at a final concentration of 100 µg/mL followed by incubation at 37 °C for 2 hours. The cells were pelleted by centrifugation and resuspended in lysis buffer (50 mM Tris-HCl at pH 7.3, 100 mM NaCl, 10% glycerol, 5 mM imidazole, 1X protease inhibitor cocktail, 0.5 mM tris(2-carboxycethyl)phosphine hydrochloride [TCEP], 0.1 mM phenylmethylsulfonyl fluoride [PMSF], 25k/µL benzonase). Cell lysis was performed using a French Press G-M (GlenMills, Clifton, NJ). Affinity purification was performed with a HisTrap HP column (Cytiva, Marlborough, MA) using the ÄKTA pure chromatography system (Cytiva). The appropriate fractions were pooled and dialyzed to PBS containing TCEP. The his-tag was cleaved at the 3C site using the Pierce HRV 3C Protease Solution Kit (Thermo Fisher Scientific) according to the manufacturer’s instructions. The cleaved his-tag was removed by nickel resin in a CrystalCruz chromatography column (Santa Cruz Biotechnology, Dallas, TX). Protein samples were concentrated using an Amicon Ultra Centrifugal Filter, 10 kDa molecular weight cut off (Millipore Sigma). Protein concentration was determined using a Pierce Bicinchoninic Acid (BCA) Protein Assay Kit (Thermo Fisher Scientific).

LRP1, alternatively known as α_2_-macroglobulin receptor in earlier literature,^19^ was purified from human placenta as previously described.^20^

### Protein gel electrophoresis, Coomassie staining, and Western blotting

Protein samples were prepared for denaturing polyacrylamide gel electrophoresis by adding NuPAGE LDS Sample Buffer (Thermo Fisher Scientific) and NuPAGE Sample Reducing Agent (Thermo Fisher Scientific) to the protein samples and heating at 95 °C for 5 minutes. The samples were loaded on a NuPage Bis-Tris Mini Protein Gel, 4%-12% (Thermo Fisher Scientific) for electrophoresis. Coomassie staining was performed with the InstantBlue Coomassie Protein Stain (Abcam, Cambridge, UK). For Western blotting, the protein transfer was performed using the Invitrogen iBlot 2 Gel Transfer Device (Thermo Fisher Scientific). Membranes were briefly washed in tris-buffered saline (TBS). Blocking was performed with Intercept Blocking Buffer (LI-COR Biosciences, Lincoln, NE). Primary antibodies were diluted in Intercept T20 Antibody Diluent (LI-COR Biosciences) and incubated at 4 °C overnight. The membrane was washed with TBS with Tween-20 (TBST) and TBS. Secondary antibodies were diluted in Intercept T20 (TBS) Antibody Diluent and incubated at room temperature for 1 hour. The membrane was washed with TBST and TBS. The membranes were imaged on an Odyssey CLx Imager (LI-COR Biosciences, Lincoln, NE).

### Tau uptake assay

CHO cells were used 1 day after plating, and iPSC-derived neurons were treated at 14  DIV. Cells were treated with 100 nM of tau proteins for 1 hour followed by a wash with PBS. Immunocytochemistry was subsequently performed.

### Image analysis

Confocal images were transformed into maximum intensity Z-projections and split into the component channels for each fluorophore using FIJI.^21^ Panels for publication were made using the EZFig plugin.^22^ Fluorescence intensity was evaluated using CellProfiler.^23^ First, the nuclei were identified by nuclear staining. Then, the cytoplasm was identified using either the tubulin staining for CHO cells or microtubule-associated protein 2 (MAP2) staining for iPSC-derived neurons. Then the fluorescence intensity of the HiBiT-tagged tau within the cytoplasm was measured. Because a Z-projection was used, nuclei were not masked to avoid excluding HiBiT-tag signal above or below the nucleus in the *Z*-axis. A custom Python script was used to average the intensities from each cell.

### Surface plasmon resonance

The binding of full-length 2N4R (splice variant containing exons, 2, 3, and 10) tau proteins with wildtype, 8D2Q, or 20D3Q sequences to LRP1 was assessed using the Biacore 8K surface plasmon resonance (SPR) system. Full-length LRP1 purified from human placenta^20^ was immobilized via amine coupling on the surface of a CM5 Biacore sensorchip. Increasing concentrations (3.7, 11.1, 33.3 nM) of recombinant tau proteins were flowed over the sensorchip in a single cycle kinetic titration experiment at pH 7.4.

### Statistical analysis

Statistical analysis was performed using Prism 10.1.2 (GraphPad, San Diego, CA). Error bars on graphs represent the standard error of the mean. A Shapiro-Wilk test was used to test for normality. For data sets comparing 2 groups with a non-normal distribution, a Mann-Whitney test was used. For data sets comparing more than 2 groups with a normal distribution, a one-way analysis of variance (ANOVA) test was used with a Šidák correction used to correct for multiple comparisons. For data sets comparing more than 2 groups with a non-normal distribution, a Kruskal-Wallis test was used in conjunction with Dunn’s test to correct for multiple comparisons. An *α*  <  0.05 or adjusted *P *<* *.05 was considered to be statistically significant.

### Diagram generation

Some diagrams were created with biorender.com, as indicated. Diagrams of protein sequences were generated with the program IBS.^24^

### Data availability

The datasets used and/or analyzed during the current study are available from the corresponding author on reasonable request. The plasmids used in the study have been deposited in Addgene, and the Addgene ID numbers are listed in Table S1.

## RESULTS

### Development of a cellular model of tau propagation

**Figure 1.**
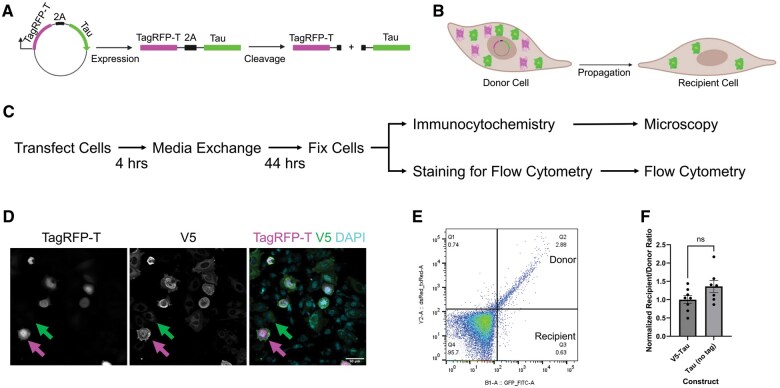
A cellular model of tau propagation. A model of tau propagation was developed for use in CHO cell culture. (A) Schematic of tau propagation construct expressing a polypeptide containing fluorescent protein TagRFP-T, a self-cleaving 2A peptide sequence, and a tau 2N4R tau sequence with or without an amino-terminal V5 tag, which is then cleaved at the 2A site into separate TagRFP-T and tau fragments. Created with biorender.com. (B) Schematic of the tau propagation experiment in the cell culture model. A transfected donor cell expresses both the TagRFP-T (magenta) and tau (green) proteins. The tau is propagated to an untransfected recipient cell, which is identified as containing tau but not TagRFP-T. Created with biorender.com. (C) Experimental overview of the tau propagation assay with either immunocytochemistry or flow cytometry as readouts. (D) Immunocytochemistry of CHO cells transfected with a tau propagation construct. Donor cells are identified by staining for TagRFP-T and V5 (example shown by magenta arrow). Recipient cells are identified by staining for V5 only (green arrow). Scale bar: 50 µm. (E) Example flow cytometry dot plot with green fluorescence intensity (V5) on the *x*-axis and red fluorescence intensity (TagRFP-T) on the *y*-axis. Each dot represents a cell. Quadrants corresponding to donor (green^+^red^+^) and recipient cells (green^+^red^-^) are indicated. (F) Comparison of tau propagation constructs with or without the amino-terminal V5 tag using flow cytometry readout demonstrating no difference with or without a V5-tag. Data analyzed by unpaired *t* test.

We developed a cellular model of tau propagation by adapting a strategy previously used in animal models,^25^ for use in a cell culture model. A polypeptide containing a red fluorescent protein (TagRFP-T), the self-cleaving 2A peptide, and the tau 2N4R protein (with or without an amino-terminal V5-tag) were cloned into a mammalian expression vector. This construct allows the expression of the polypeptide, which undergoes self-cleavage at the 2A site to produce separate TagRFP-T and tau proteins (Figure 1A). Expression of this tau propagation construct in a suitable cell line allows the identification of donor cells expressing the polypeptide, which contains both the separate TagRFP-T and tau components, and recipient cells, which contain tau as a result of tau propagation but do not contain TagRFP-T (Figure 1B). For transient transfections in this experiment, the transfection efficiency is intentionally kept low (∼20%) to allow for a large pool of untransfected potential recipient cells. Chinese hamster ovary cells were chosen as the cell line for these experiments because they are easily transfected and express LRP1,^26^ which is a known receptor for tau uptake.^10^^,^^11^ For the tau propagation experiments, the protocol involved a transient transfection of the CHO cells with a tau propagation construct, followed by a media exchange after 4 hours in order to remove the transfection reagent from the media to reduce the risk of the transfection reagent interfering with the tau propagation. Two assays were used to demonstrate tau propagation in this system, that is, immunocytochemistry or flow cytometry (Figure 1C). For the immunocytochemistry experiments, the cells were stained with a V5-antibody to detect V5-tau and counterstained with DAPI. TagRFP-T retained its fluorescence with the immunocytochemistry protocol and did not require antibody detection. Donor cells were identified as containing TagRFP-T signal and V5 signal (magenta arrow, Figure 1D), and recipient cells were identified as containing V5 signal without TagRFP-T signal (green arrow, Figure 1D). As expected, essentially no cells were observed with TagRFP-T signal but without V5 signal. The flow cytometry experiment was performed by fixing, permeabilizing, and staining the cells with a V5 antibody conjugated to Alexa Fluor 488. This green fluorescent signal, along with the red fluorescent signal of the TagRFP-T was used to identify cells in a flow cytometer. The donor cells are TagRFP-T (red)-positive and V5 (green)-positive, whereas the recipient cells are TagRFP-T (red)-negative and V5 (green)-positive (Figure 1E). In order to confirm that the V5 tag on the amino-terminus of tau did not impact tau propagation, a tau propagation construct lacking the V5 tag was compared to the construct containing the V5 tag. For this experiment, tau propagation was determined by comparing the ratio of recipient/donor cells for both constructs using flow cytometry. Since the V5 tag was absent from one construct, a human tau (HT7) antibody conjugated to Alexa fluor 488 was used to detect tau from both constructs. There was no significant difference in the tau propagation using these constructs (Figure 1F), suggesting that the V5 tag does not impact tau propagation in this assay. Given this, the V5 tag was included on the amino-terminus of tau in future constructs to allow for easy detection of tau derived specifically from the tau propagation construct.

### Tau PTM mimics alter tau propagation

**Figure 2.**
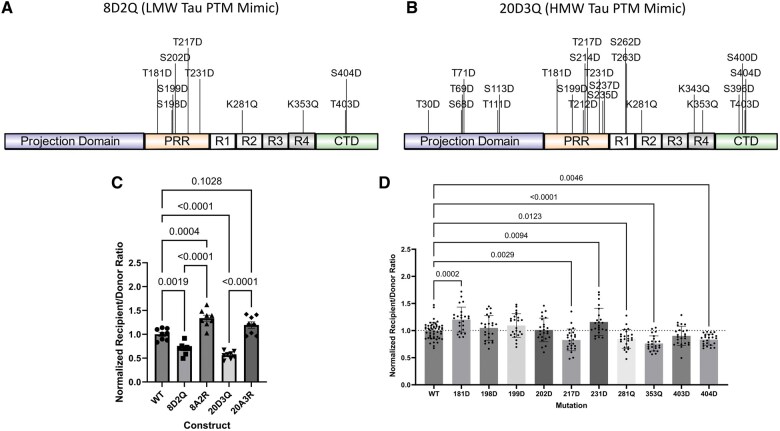
Alzheimer disease-associated tau PTM mimics influence tau propagation. Tau propagation constructs containing AD-associated tau PTM mimics or the wildtype tau sequence were compared in the cellular model of tau propagation using a flow cytometry readout. (A) Diagram of mutations in the 8D2Q construct that mimics the PTMs found in LMW tau in AD patients. Diagram made with IBS.^24^ (B) Diagram of mutations in the 20D3Q construct that mimics the PTMs found in HMW tau in AD patients. Diagram made with IBS.^24^ (C) Comparison of wildtype tau, AD-associated tau PTM mimics, or mutations that impair AD-associated PTMs demonstrating the tau PTM mimics reduced tau propagation, while the mutations that impair AD-associated PTMs enhanced tau propagation compared to wildtype. Data analyzed by one-way ANOVA. (D) Comparison of wildtype tau and individual residue PTM mimics corresponding to the 8D2Q construct. The mutations with significantly increased or decreased tau propagation relative to wildtype are demonstrated with the corresponding *P*-value. Data analyzed by one-way ANOVA.

In order to assess the impact of AD-associated tau PTMs on tau propagation in this cellular model, we developed a series of tau propagation constructs that use genetically encoded amino acids to mimic the charge changes associated with PTMs on AD-associated tau. To mimic the additional negative charge associated with phosphorylation of serine or threonine residues, a mutation of these residues to aspartate was used.^27^ The neutralization of the positive charge of lysine by acetylation was mimicked by mutating lysine to glutamine.^28^ The residues of interest for modification were identified as AD-associated PTMs previously defined on LMW and HMW tau species found in postmortem AD brain tissue.^17^ The LMW tau species contained a total of 8 phosphorylations and 2 acetylations,^17^ so the resultant mutant tau sequence to mimic these changes contained 8 aspartate (D) mutations and 2 glutamine (Q) mutations, which is abbreviated as the 8D2Q construct (Figure 2A). The HMW tau species contained a total of 20 phosphorylations and 3 acetylations,^17^ so the resultant mutant tau sequence contained 20 aspartate and 3 glutamine mutations, which is abbreviated as the 20D3Q construct (Figure 2B). Additional constructs were made with mutations at the same residues that prevent posttranslational modification at that residue. To prevent phosphorylation, serine (S) or threonine (T) was mutated to alanine (A), and to prevent acetylation, lysine (L) was mutated to arginine (R). These constructs were designated as 8A2R and 20A3R to correspond to the residues with PTMs in LMW and HMW tau, respectively. These tau propagation constructs, along with the construct containing the WT tau sequence, were used in the CHO cell model of tau propagation with the flow cytometry readout (Figure 1C). Compared to the WT construct, both the 8D2Q and the 20D3Q constructs showed a significant reduction in propagation (Figure 2C). In contrast, the 8A2R construct showed a significant increase in propagation compared to the WT construct. The 20A3R construct also showed a trend toward increased propagation compared to the WT construct, though this did not reach statistical significance (Figure 2C). Both the 8D2Q and 20D3Q construct had significantly reduced propagation compared to the corresponding construct that does not permit post-translational modification at the same residues (Figure 2C).

Since the 8D2Q and 20D3Q constructs contain mutations at multiple residues, we next sought to understand how PTM mimics at individual residues might impact tau propagation. Since the tau propagation seen with the 8D2Q construct is similar to that of the 20D3Q construct but has less than half the number of mutated residues, we made a series of tau propagation constructs based on the 8D2Q construct that contain one PTM mimic mutation each per construct. We tested these constructs in comparison with the WT construct in the CHO cell model of tau propagation with the flow cytometry readout (Figure 1C). Among these ten constructs, the T181D and T231D mutant constructs had significantly increased tau propagation compared to WT, the T217D, K281Q, K353Q, and T404D mutant constructs had significantly decreased tau propagation compared to WT, and the remaining constructs did not show a significantly different degree of tau propagation compared to WT (Figure 2D). While the mutations at the individual residues had different impacts on tau propagation compared to WT, 4 residues reduced tau propagation but only 2 residues enhanced it. This may be why the net effect of all the mutations in the 8D2Q construct is to reduce tau propagation.

### Tau PTM mimics do not impact tau levels in media

**Figure 3.**
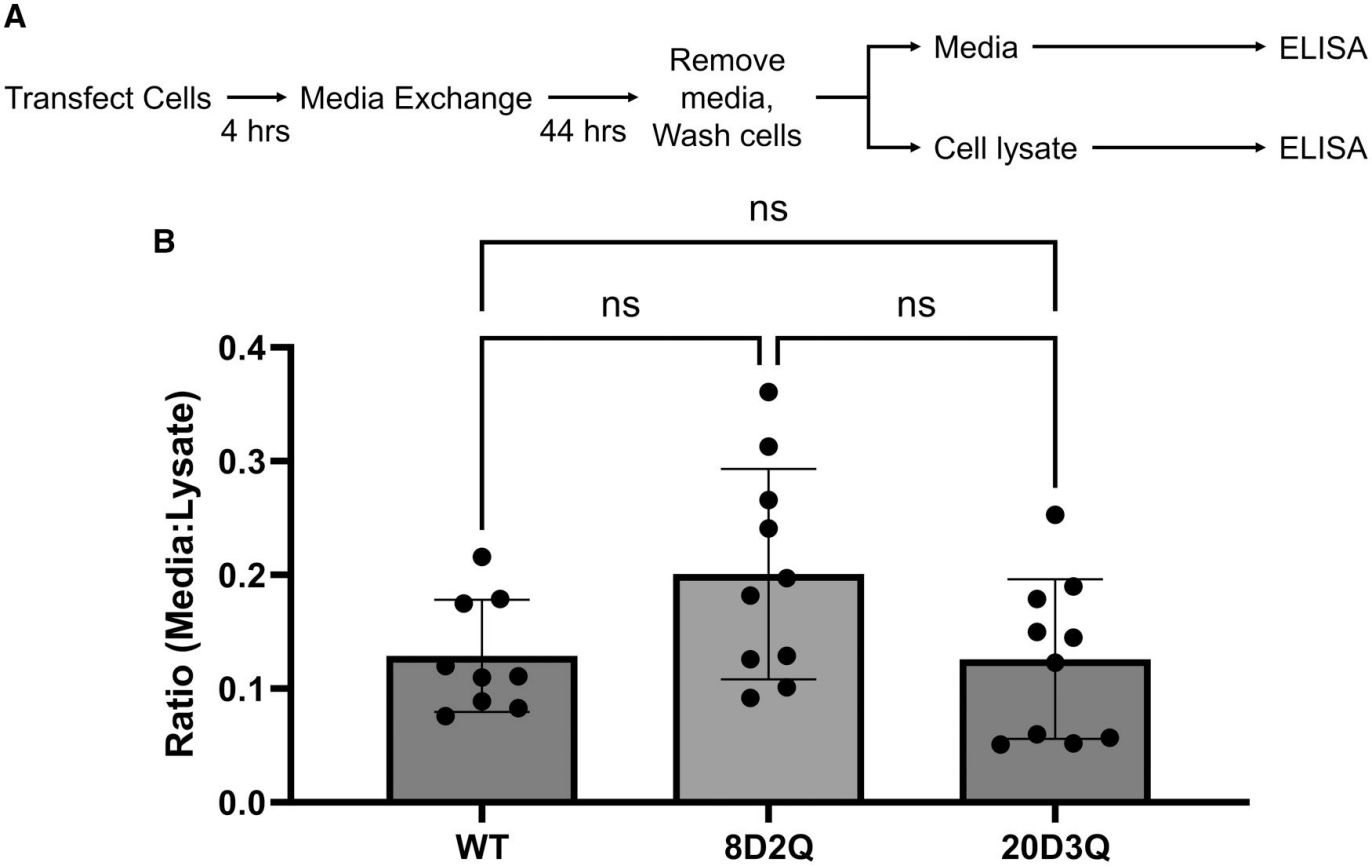
Alzheimer disease-associated tau PTM mimics do not impact tau release from donor cells. Tau propagation constructs AD-associated tau PTM mimics or the wildtype tau sequence were compared in the cellular model of tau propagation with a tau immunoassay as the readout. (A) Schematic of the experimental design. (B) Comparison of wildtype tau or AD-associated tau PTM mimics demonstrating no significant differences in release from wildtype or mutant constructs. Data analyzed by one-way ANOVA.

The cell-to-cell propagation of tau can be broken down into 2 steps: the release of tau from the donor cell and the uptake of tau by the recipient cell. To assess the potential impact of tau PTM mimics on tau release by donor cells, we used the tau propagation constructs in a similar CHO cell assay, but with a read-out of a tau ELISA (Figure 3A). The tau ELISA was used to identify tau in the cell media in as a steady state measure of extracellular tau and the cell lysate as a control for transfection efficiency. With this assay, there was no significant difference in tau in the media comparing WT and tau PTM mimic constructs (Figure 3B). This suggests that differences in tau propagation are not consequent to the extracellular tau levels as expressed by donor cells.

### Tau PTM mimics impact tau uptake in CHO cells

**Figure 4.**
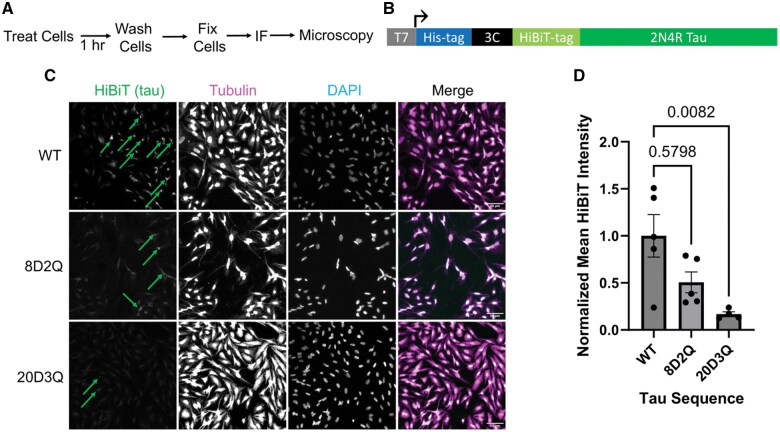
Alzheimer disease-associated tau PTM mimics reduce tau uptake in CHO cells. Tau uptake was assessed in CHO cells using an immunocytochemistry assay. (A) Schematic of the experimental design. (B) Diagram of protein expression construct open reading frame used to express wildtype or mutant tau proteins. (C) Representative images of immunocytochemistry of CHO cells treated with wildtype or AD-associate tau PTM mimic proteins. Scale bar: 50 µm. The green arrows indicate areas of qualitatively bright signal in the HiBiT (tau) channel. (D) Comparison of tau uptake of wildtype or AD-associate tau PTM mimic proteins in CHO cells demonstrating a trend toward reduced uptake of mutant proteins compared to wildtype. Data analyzed by Kruskal-Wallis test.

Next, we examined the potential impact of tau PTM mimics on tau uptake by recipient cells by observing uptake of recombinant tau, thus isolating uptake from release-associated mechanisms. For this, CHO cells were treated with recombinant tau proteins for 1 hour, washed 3 times, fixed, and subjected to immunofluorescence (IF) to allow for microscopy and fluorescent intensity quantification (Figure 4A). The bacterial expression construct for the recombinant tau included a his-tag for affinity purification, a 3C cleavage site to remove the his-tag after purification, a HiBiT-tag that serves as a convenient marker on the amino-terminus of tau, and the full-length 2N4R tau sequence, with or without the tau PTM mimic mutations (Figure 4B). Of note, the mutations that prevent PTM (eg, in the 8A2R and 20A3R constructs) were not included in the experiments with recombinant tau proteins because their effect would be to prevent PTMs from being introduced at the mutated residues. However, the *E. coli* used for expression of recombinant proteins do not produce post-translationally modified proteins, so the effect of these mutations would be expected to be minimal compared to the wildtype tau protein, which would have no PTMs on it. The recombinant tau proteins were expressed and purified by affinity purification followed by his-tag cleavage (Figure S1). The recombinant tau proteins with the WT, 8D2Q, and 20D3Q tau sequences were used to treat CHO cells in an imaging-based tau uptake experiment (Figure 4A). The IF was performed with an anti-HiBiT antibody as a marker for the HiBiT-tau protein and an anti-tubulin antibody as a cytoplasmic marker and a nuclear counterstain to identify nuclei. Visual inspection of the images generally suggested that the 8D2Q and 20D3Q proteins had reduced uptake compared to the WT tau sequence (Figure 4C). The fluorescence intensity of the anti-HiBiT antibody within the cytoplasm as defined by the anti-tubulin antibody signal was quantified using CellProfiler. The comparison between WT and 20D3Q revealed a statically significant difference, with 8D2Q having an intermediate level (Figure 4D). These data suggest that the tau PTM mimics, particularly those found in the 20D3Q mutant, reduce tau uptake.

### Tau PTM mimics impact tau uptake in iPSC-derived neurons

**Figure 5.**
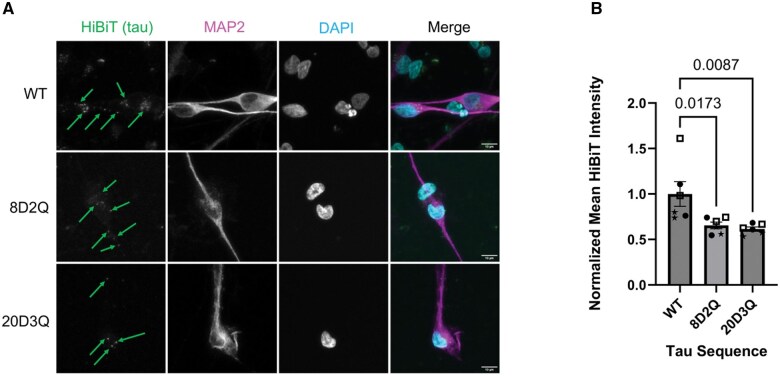
Alzheimer disease-associated tau PTM mimics reduce tau uptake in iPSC-derived neurons. Tau uptake was assessed in iPSC-derived neurons using an immunocytochemistry assay. (A) Representative images of immunocytochemistry of iPSC-derived neurons treated with wildtype or AD-associate tau PTM mimic proteins. Scale bar: 10 µm. The green arrows indicate areas of qualitatively bright signal in the HiBiT (tau) channel. (B) Comparison of tau uptake of wildtype or AD-associate tau PTM mimic proteins in iPSC-derived neurons demonstrating significantly reduced uptake of mutant proteins compared to wildtype. Data from each iPSC line are represented by a different symbol. Data analyzed by one-way ANOVA.

Although the CHO cell system serves as a convenient model for investigating tau propagation and uptake, a more relevant cell model for AD is an iPSC-derived neuron culture. As such, we adapted the tau uptake assay (Figure 4A) for an iPSC-derived neuron model. Three independent iPSC lines (derived from 3 different individuals) were used in the experiment. The iPSCs were differentiated to neurons using *hNGN2* expression with a previously published method.^18^ The iPSC-derived neurons were treated with the recombinant tau proteins in the imaging-based tau uptake assay (Figure 4A). The IF staining was similar to that performed in CHO cells, except that MAP2 was used as the cytoplasmic marker instead of tubulin. The visual pattern of reduced tau uptake of recombinant 8D2Q and 20D3Q tau proteins was seen in the IF images (Figure 5A). Quantification of the tau fluorescent signal in CellProfiler revealed a significant reduction in tau uptake for both the 8D2Q and 20D3Q tau proteins compared to the WT tau protein using iPSC-derived neurons (Figure 5B). These data suggest that the tau PTM mimics reduce tau uptake in human iPSC-derived neurons.

### Tau PTM mimics have reduced binding to LRP1

**Figure 6.**
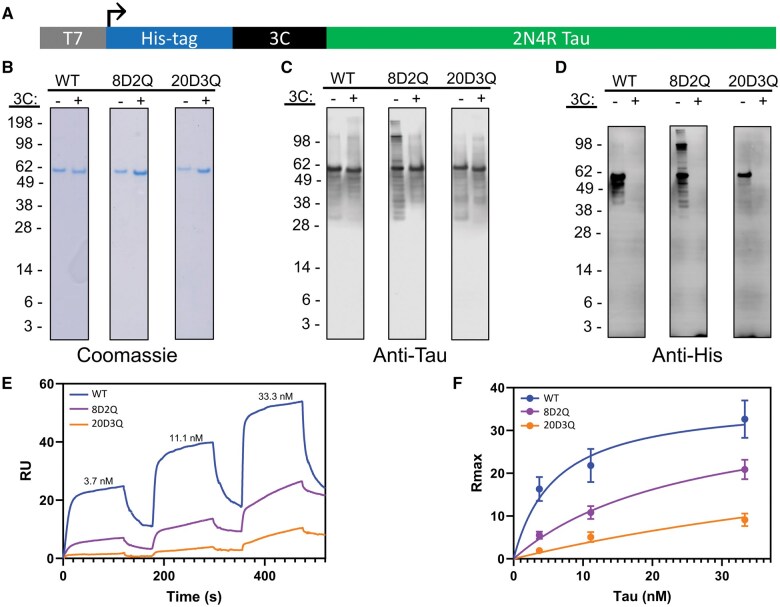
Alzheimer disease-associated tau PTM mimics have reduced affinity for LRP1. Determination of the affinity of AD-associated tau PTM mimic proteins for LRP1. (A) Diagram of protein expression construct open reading frame used to express wildtype or mutant tau proteins. (B) Gel electrophoresis of expressed proteins with or without the his-tag removed by 3C cleavage stained with Coomassie stain. (C) Western blot of expressed proteins with or without the his-tag removed by 3C cleavage using an anti-tau antibody. (D) Western blot of expressed proteins with or without the his-tag removed by 3C cleavage using an anti-his-tag antibody. (E) Representative images of a single surface plasmon resonance experiment. (F) Nonlinear regression analysis of *R*_max_ vs concentration of surface plasmon resonance experiments demonstrating reduced affinity of tau mutants for LRP1 compared to wildtype tau.

Since LRP1 is a known receptor for tau uptake and since lysine are critical for LRP1-tau interactions,^10^^,^^11^ we hypothesized that the reduced uptake of tau PTM mimics could be due to decreased affinity for LRP1. In order to test this hypothesis, we developed bacterial expression constructs for full-length 2N4R tau (Figure 6A) with the WT, 8D2Q, and 20D3Q tau sequences. This expression construct is similar to the one used previously (Figure 4A), but this construct lacks the amino-terminal HiBiT-tag to avoid any potential interference with binding caused by the HiBiT-tag. The recombinant proteins were expressed, purified by affinity chromatography with the his-tag, and the his-tag was removed by 3C cleavage. In order to evaluate the quality of the proteins produced, the protein samples were subjected to denaturing polyacrylamide gel electrophoresis followed by Coomassie staining. For each tau protein sequence, the his-tag-containing protein (prior to 3C cleavage) and the protein without the his-tag (after 3C cleavage) were included. For each of the tau proteins, a single band is identified between the 62 and 49 kDa markers. The proteins for which the his-tag has been cleaved (by addition of the HRV 3C protease) run subtly lower than those containing the his-tag, suggesting that the his-tag was successfully cleaved (Figure 6B). To further confirm this, a Western blot of the protein samples was performed using anti-tau and anti-his antibodies. The Western blot probing for tau showed a major band between the 62 and 49 kDa markers (Figure 6C), corresponding to the band seen on the Coomassie stain (Figure 6B). Minor bands were also seen below the major tau band (Figure 6C), likely reflecting a small degree of degradation despite the inclusion of protease inhibitors. The anti-his antibody demonstrated a major band between the 62 and 49 kDa markers as well, but this staining is present only in the samples without 3C cleavage (Figure 6D), indicating that the 3C cleavage of the his-tag was successful. Binding of the WT, 8D2Q, and 20D3Q recombinant tau proteins to LRP1 was assessed using SPR. Increasing concentrations (3.7, 11.1, 33.3 nM) of recombinant 2N4R, 20D3Q, or 8D2Q tau were flowed over a sensorchip with LRP1 in a single cycle kinetic titration experiment at pH 7.4 (Figure 6E). Nonlinear regression of *R*_max_ vs concentration revealed both the 8D2Q and 20D3Q recombinant tau proteins had reduced binding affinity for LRP1 compared to the WT tau protein. The apparent *K*_D_ values for the WT, 8D2Q, and 20D3Q tau proteins derived from the regressions were 6, 26, and 93 nM, respectively.

## DISCUSSION

The development of a cellular model (Figure 1) for easily studying tau propagation facilitates mechanistic dissection of the processes involved. A similar experimental paradigm using related reporter constructs has been utilized in vivo to study tau propagation in mice.^10^^,^^16^^,^^25^^,^^29^ The use of the cellular model allows for evaluation of a relatively large number of constructs that would likely be impractical to use with in vivo studies. Additionally, cell models allow for examination of the individual steps involved in tau propagation to provide more mechanistic insights into this process.

In the CHO cell model of tau propagation, AD-associated PTM mimics (Figure 2A and B) led to reduced tau propagation compared to WT tau (Figure 2C), suggesting the PTMs found on LMW and HMW tau species in AD likely have analogous effects. Conversely, mutations that prevent PTM at those residues showed a trend toward increased tau propagation compared to WT tau (Figure 2C), which can undergo PTM in cellulo. Examining the effect of individual PTM mimics within the 8D2Q construct revealed variable impacts on propagation, with some pseudophosphorylations increasing propagation and others reducing it but with pseudoacetylation consistently reducing tau propagation (Figure 2D).

Tau phosphorylation states have previously been shown to influence tau propagation. In a mouse model, compared to tau preparations from AD brains, similar tau preparations that had undergone in vitro dephosphorylation have been shown to reduce tau propagation.^14^ In our study, pseudophosphorylation had variable impacts on tau propagation (Figure 2D), and this variability may not have been reflected in the in vivo study because it used dephosphorylation of multiple sites at a time,^14^ rather than specifying changes at individual residues. In a *Drosophila melanogaster* cell culture model, pseudophosphorylation mimics of hyperphosphorylated tau increased tau secretion.^30^ In contrast, our study using CHO cells revealed no change in tau release into the media (Figure 3B). This may be because cell lines from different phyla were used or because the combinations of PTM mimics used were different, impacting effects on tau behavior. A prior study has shown that tau phosphorylation reduced tau uptake in 3 different non-neuronal cell lines.^11^ This study revealed that tau constructs containing pseudophosphorylations had reduced uptake in both non-neuronal and neuronal cell types (Figures 4C, D and 5A, B).

Tau acetylation has also been shown to impact processes related to tau propagation. Lysine acetylation is particularly relevant for tau uptake since LRP1-mediated tau uptake is dependent on tau lysines such that chemically capping these lysines prevented tau uptake.^10^ In line with this finding, pseudoacetylations at several residues in tau have also been shown to decrease tau uptake.^11^ This is concordant with the results of our study, which also showed that tau uptake is reduced for tau proteins containing pseudoacetylations (Figures 4C, D and 5A, B). These tau proteins also have reduced affinity for LRP1 (Figure 6E and F), which may explain, at least in part, the reason for the reduced tau uptake. The reduced uptake of tau with AD-associated PTM mimics may be responsible for the reduced propagation seen with these constructs (Figure 2C and D).

Our findings indicate that tau containing AD-associated PTM mimics have reduced uptake by recipient cells. These data help distinguish the differential impact of AD-associated and pathogenic PTMs on the uptake of extracellular tau in CHO cells and iPSC-derived neurons. In general, unmodified tau is an excellent LRP1 substrate, yet even the modest number of PTMs present on physiologic LMW tau isolated from normal human brain diminish uptake, and the larger number of PTMs present on seed-competent tau does so even more. The finding that tau uptake, and therefore cell-to-cell propagation, is reduced for the tau proteins containing AD-associated PTM mimicking mutations may seem somewhat counterintuitive given that tau propagation is a feature of AD pathophysiology. However, the release of tau by donor cells and uptake of tau by recipient cells represent only a subset of the processes required for the successful propagation of pathological tau. Additional steps are required, such as escape from the endolysosomal system and templated aggregation of the endogenous tau of the recipient cell,^6^ but these steps were not assessed in this study and represent areas that require additional study in future experiments. Overall, the results of this study suggest that intracellular aggregation and seeding rather than release and/or uptake of tau may be the rate-limiting step in tau propagation in AD. This possibility could explain the apparent contradiction between the results of this study and the fact that tau propagation is a key aspect of AD. Tau PTMs at specific residues have been found to correlate with the ability of AD-derived tau preparations to seed tau aggregation.^31^ The acetylation of lysine residues have particularly been shown to enhance the aggregation propensity of tau.^32–34^ Thus, we speculate that although the cell-to-cell propagation of tau with AD-associated PTMs may be less robust than the propagation of tau with fewer PTMs, once highly aggregation-prone tau with AD-associated PTMs is taken up in a recipient cell, it is sufficient to template the aggregation of tau in the recipient cell and further promote propagation of pathologic tau to other cells.^6^

The results of this study may help partially provide a rationale for clinically observed phenomena. Phosphorylated tau, particularly at residues 181 and 217, is elevated in both cerebrospinal fluid and plasma, which allows them to serve as biomarkers for AD diagnosis.^35^ Our study has shown that tau with AD-associated tau PTM mimics, including at residues 181 and 217, has impaired uptake into recipient cells (Figures 4D and 5B). Since release by donor cells is not affected by the PTM mimics (Figure 3B), this would lead to increased interstitial accumulation of the tau with AD-associated PTMs. This could eventually lead to increased levels of tau with these PTMs in the cerebrospinal fluid and plasma and may explain, at least in part, why phosphorylated tau at residues 181 and 217 are elevated in biofluids in AD.

While this study provides some insights into the role of tau PTMs in influencing tau propagation, our study does have some limitations. We used genetically encoded PTM mimics in this study, rather than the actual PTMs. While the PTM mimics alter charge similarly to the actual PTMs, the exact chemical structures differ. In some instances, this may result in differential behavior between the PTMs and PTM mimics. Our approach of using cell culture models allows for easy manipulation of the system, but it does lack the complexity of in vivo systems. For example, potential effects of glial cells on tau propagation^36^^,^^37^ are not included in our cell culture models. Extension of these studies into animal models in the future could provide a more comprehensive evaluation of the impact of tau PTMs on tau propagation in an intact brain with all cell types represented. Despite these limitations, this investigation has revealed mechanistic insights into the role that specific AD-associated PTMs may play in influencing tau propagation.

Overall, this study used cellular models to study the impact of AD-associated tau PTMs on tau propagation and the mechanisms that underlie this process, such as release from donor cells and uptake by recipient cells. We found that AD-associated tau PTM mimics reduce tau propagation through reduced uptake by recipient cells rather than reduced release by donor cells. This reduced uptake is likely due in part to reduced affinity for the tau uptake receptor LRP1. These findings enhance our understanding of pathophysiological processes involved in AD progression.

## Supplementary Material

nlaf007_Supplementary_Data
