# Supplementary material for: Alzheimer disease-associated tau post-translational modification mimics impact tau propagation and uptake
Source: J Neuropathol Exp Neurol. 2025 Feb 21;84(6):459–70. doi: 10.1093/jnen/nlaf007 (PMC12096005; doi:10.1093/jnen/nlaf007)
Supplement: nlaf007_Supplementary_Data [file nlaf007_supplementary_data.pdf]

Alzheimer disease-associated tau post-translational modification mimics impact tau propagation and uptake

Running title: AD-associated tau PTMs impact tau propagation

John R. Dickson, MD, PhD[1,2], Robert G.R. Sobolewski, BS[1], Analiese R. Fernandes, BS[1,3,4], Joanna M. Cooper, PhD[5,6], Zhanyun Fan, MSc[1], Mirra Chung, MS[1], Cameron Donahue, BS[1,7], Derek H. Oakley, MD, PhD[2,8], Dudley K. Strickland, PhD[5,6,9], Bradley T. Hyman, MD, PhD[1,2,*]

[1]Alzheimer Research Unit, Department of Neurology, Massachusetts General Hospital, Charlestown, Massachusetts, USA; [2]Faculty of Medicine, Harvard Medical School, Boston, Massachusetts, USA; [3]Present Address: Mayo Clinic Graduate School of Biomedical Sciences, Jacksonville, Florida, USA and [4]Department of Neuroscience, Mayo Clinic, Jacksonville, Florida, USA; [5]The Center for Vascular and Inflammatory Diseases, University of Maryland School of Medicine, Baltimore, Maryland, USA; [6]Department of Physiology, University of Maryland School of Medicine, Baltimore, Maryland, USA [7]Present address: Graduate Program for Neuroscience, Boston University, Boston, Massachusetts, USA; [8]C.S. Kubik Laboratory for Neuropathology, Department of Pathology, Massachusetts General Hospital, Boston, Massachusetts, USA; [9]Department of Surgery, University of Maryland School of Medicine, Baltimore, Maryland, USA. *Corresponding author – address: CNY B114-2-2003, 114 16th Street, Charlestown, MA 02129; email address: bhyman@mgh.harvard.edu

Supplemental Data

Contents

Figure S1: Page S-2

Table S1: Page S-3

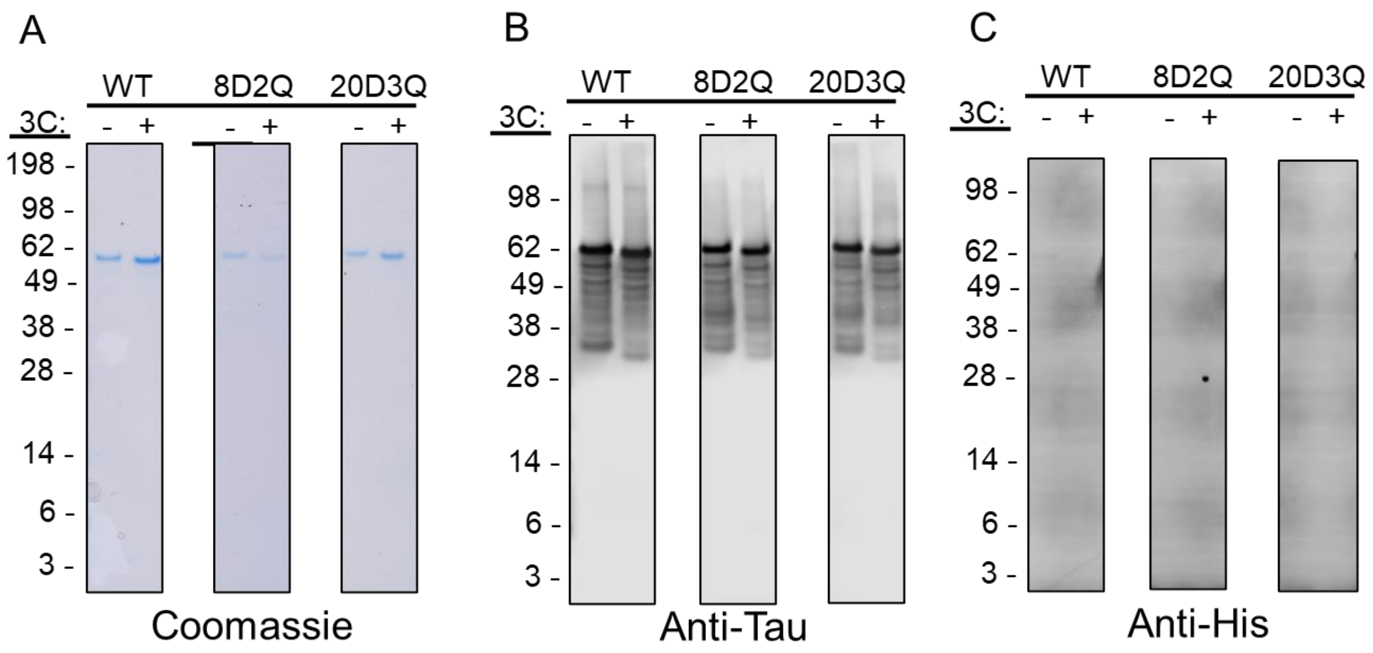

Figure S1. Expression and cleavage of HiBiT-tagged tau proteins.

HiBiT-tagged tau proteins were expressed in *E. coli* and cleaved at the 3C cleavage site after affinity chromatography. (A) Gel electrophoresis of expressed and cleaved or uncleaved proteins stained with Coomassie stain. (D) Western blot of expressed and cleaved or uncleaved proteins using an anti-tau antibody. (E) Western blot of expressed and cleaved or uncleaved proteins using an anti-his-tag antibody.

Table S1. Plasmids with Addgene IDs.

Plasmid	Addgene ID
pcDNA3.1(-).TagRFP.T-2A-Tau2N4R-WPRE	226380
pcDNA3.1(-).TagRFP.T-2A-V5-Tau2N4R-WPRE	226381
pcDNA3.1(-).TagRFP.T-2A-V5-8D2Q.Tau2N4R-WPRE	226382
pcDNA3.1(-).TagRFP.T-2A-V5-20D3Q.Tau2N4R-WPRE	226383
pcDNA3.1(-).TagRFP.T-2A-V5-8A2R.Tau2N4R-WPRE	226384
pcDNA3.1(-).TagRFP.T-2A-V5-20A3R.Tau2N4R-WPRE	226385
pcDNA3.1(-).TagRFP.T-2A-V5-K281Q.Tau2N4R-WPRE	226386
pcDNA3.1(-).TagRFP.T-2A-V5-K353Q.Tau2N4R-WPRE	226387
pcDNA3.1(-).TagRFP.T-2A-V5-S198D.Tau2N4R-WPRE	226388
pcDNA3.1(-).TagRFP.T-2A-V5-S199D.Tau2N4R-WPRE	226389
pcDNA3.1(-).TagRFP.T-2A-V5-S202D.Tau2N4R-WPRE	226390
pcDNA3.1(-).TagRFP.T-2A-V5-S404D.Tau2N4R-WPRE	226391
pcDNA3.1(-).TagRFP.T-2A-V5-T217D.Tau2N4R-WPRE	226392
pcDNA3.1(-).TagRFP.T-2A-V5-T403D.Tau2N4R-WPRE	226393
pcDNA3.1(-).TagRFP.T-2A-V5-T181D.Tau2N4R-WPRE	226394
pcDNA3.1(-).TagRFP.T-2A-V5-T231D.Tau2N4R-WPRE	226395
pET28.His.3C.Tau2N4R	226396
pET28.His.3C.20D3Q.Tau2N4R	226397
pET28.His.3C.8D2Q.Tau2N4R	226398
pET28.His.3C.86b.Tau2N4R	226399
pET28.His.3C.86b.8D2Q.Tau2N4R	226400
pET28.His.3C.86b.20D3Q.Tau2N4R	226401
